# Potential Benefits on Impairment of Endothelial Function after a High-Fat Meal of 4 Weeks of Flavonoid Supplementation

**DOI:** 10.1093/ecam/nen048

**Published:** 2011-06-05

**Authors:** T. A. Barringer, L. Hatcher, H. C. Sasser

**Affiliations:** Center for Cardiovascular Health (TAB, LH) and Dickson Institute for Health Studies (HCS), Carolinas Healthcare System, P.O. Box 32861, Charlotte, NC 28232, USA

## Abstract

Studies with foods high in flavonoids have demonstrated improvement in endothelial function. We investigated whether 4 weeks of flavonoid supplementation would prevent an adverse impact on endothelial function of a high-fat meal. Endothelial function was measured by reactive hyperemia peripheral arterial tonometry (RH-PAT). The RH-PAT index was measured both before and 3 h after a high-fat meal, in 23 healthy volunteers. Subjects were randomized in a double-blind, cross-over design to 4 weeks of daily supplementation with OPC-3, or a matching placebo. RH-PAT index before and after the high-fat meal was measured at the beginning and end of each 4-week treatment phase. The high-fat meal caused a decline in endothelial function at baseline in the placebo (*-*10.71%, *P* = .006) and flavonoid [*-*9.97% (*P* = .077)] groups, and there was no difference in decline between arms (*P* = .906). The high-fat meal produced a decline after 4 weeks of placebo [*-*12.37% (*P* = .005)], but no decline after 4 weeks of flavonoid supplement [*-*3.16% (*P* = .663)], and the difference between the two responses was highly significant (*P* < .0001). Within-group comparisons revealed no difference in endothelial function decline in the placebo arm between baseline and 4 weeks [*-*10.71% versus *-*12.37% (*P* = .758)]. In the flavonoid supplement arm, the difference in endothelial function decline between baseline and 4 weeks was *-*9.97% versus *-*3.16%, but did not reach statistical significance (*P* = .451). These results suggest that the flavonoid supplement used in this study mitigates the impairment of endothelial function caused by a high-fat meal. Whether certain subpopulations derive greater or lesser benefit remains unclear.

## 1. Introduction

A high intake of fruit and vegetables has been associated with a reduced risk for cardiovascular disease [1]. While the exact mechanisms for this benefit are unknown, there is general consensus within the scientific literature that it is at least partially derived from the antioxidant effects of the numerous polyphenols found in the diet [2–6]. In particular various foods high in flavonoids (e.g. tea, cocoa, wine, citrus fruit) have been associated with lower risk for cardiovascular disease in most, but not all, studies [7–11]. In order to determine the cause(s) of the epidemiological inconsistencies, as well as to elucidate the mechanism by which foods high in flavonoids might exert a cardiovascular protective effect, further studies in humans and animals are needed. Most importantly studies are needed using experimental designs which reduce the variability of flavonoid exposure. Such variability is an inherent problem in epidemiologic studies which estimate flavonoid content from food consumption patterns [12].

Another critical issue in experimental design, lacking in many previous studies, is the need for endpoints which are valid and reliable markers of cardiovascular health. Endothelial function is considered one of the best indicators of vascular health, and dysfunction can be viewed as the common pathway between coronary risk factors and the development of atherosclerosis [13–17]. Measurement of brachial artery dilatation after flow-mediated dilation has been the standard method for assessing endothelial function, but in recent years an equally reliable, but less operator-dependent, non-invasive test for endothelial function has been developed for use in clinical research [18–21].

Studies using nutritional supplements with precise amounts of specific flavonoids, and which measure effects on a robust marker of cardiovascular health, such as endothelial function, are sparse, and results highly variable [21, 22]. Two studies serve as examples: Hale et al. found no benefit on endothelial function after 2 weeks of supplementation with the soy flavonoid genistein in a group of 29 healthy postmenopausal women [23]. Engler et al. found that a flavonoid-rich dark chocolate (213 mg procyanidins, 46 mg epicatechin) improved endothelial function as compared to a low-flavonoid chocolate [24]. These discrepant results highlight the need for more well-designed trials using different flavonoid compounds and quantities.

Studies which use commercially manufactured supplements have an additional benefit to the consumer interested in distinguishing among the numerous botanical, holistic products available to them, all claiming salutary effects. While proof of a specific beneficial physiologic effect does not translate into evidence for better health or disease reduction, the vast majority of such products in the marketplace have not been evaluated in a single scientifically reputable study.

In an effort to address these issues we performed a study using a supplement, OPC-3 (nutraMetrix, Division of Market America, Inc.), consisting of oligomeric proanthocyanidins, anthocyanins and other phenolic acids derived from grape seed, pine bark, bilberry, citrus and red wine extracts, to determine effects on endothelial function in a group of healthy volunteers.

## 2. Subjects and Methods

Twenty-five participants were recruited from the employee population of the hospital and related clinics. All participants were at least 18 years of age, in relatively good health, and taking no medication known to impact the outcome parameters of the study. Potential enrollees were excluded for history of: coronary artery disease, cerebrovascular disease, heart failure, diabetes, renal impairment, uncontrolled hypertension, untreated or clinically evident thyroid disease, tobacco use or pregnancy or breastfeeding. Female participants also were required to use effective birth control during the study. Participants also were instructed not to change their routine diet, exercise or weight while in the study. The project was approved by Carolinas Medical Center's Institutional Review Board (IRB), and all participants signed an approved informed consent document.

Using a computer-generated list, each participant was assigned to receive in random sequence one four-week course each of the supplemental intervention and placebo. Thus each participant served as his or her own control. A 2-week wash-out period separated the two study periods. The active agent and placebo were provided as powders in identical-looking, sealed foil envelopes. Participants were instructed to consume the contents of one envelope, dissolved in 120 ml of tap water, once daily.

Each dose of the OPC-3 supplement formulation used in this study provided 250 mg of total dried extracts derived from the sources listed above. A base of inactive ingredients, including fructose, glucose, citric acid, potassium bicarbonate, silica, calcium sulfate and pectin, contributed to the tonicity of the solution once water was added, acted as a pH buffer, and covered the bitter and astringent tastes of the extracts to improve the uniformity of powder blending. The placebo was comprised of fructose, citric acid, potassium bicarbonate, maltodextrin, silica, calcium sulfate, apple fiber, FD&C Red #40 and FD&C Blue #1.

**Figure 1:**
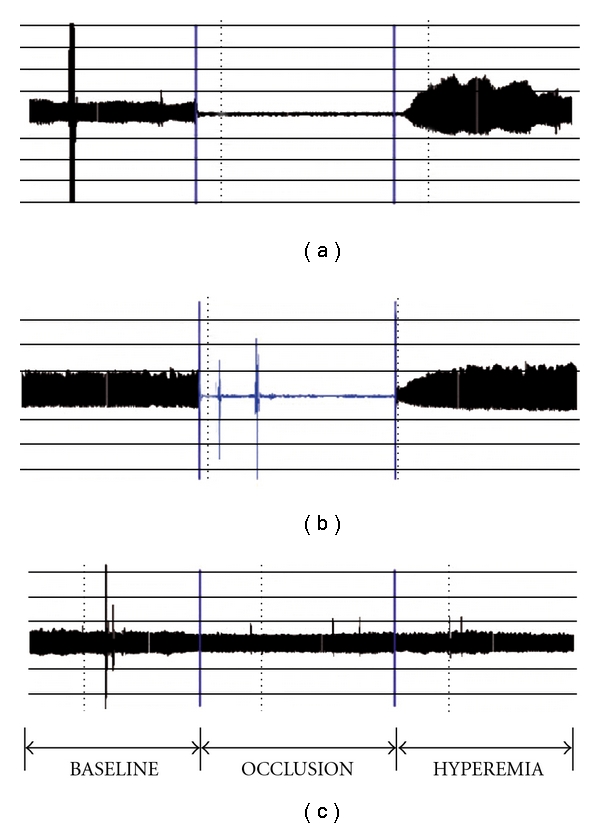
(a) Digital pulse volume recording of an individual in the fasting state showing a steady-state PAT at baseline, complete disappearance of the signal during cuff inflation (occlusion), followed by an increased PAT signal during recovery (hyperemia phase). (b) Recording in same individual obtained 3 h after a high-fat meal showing a blunted finger PAT response during the reactive hyperemia phase. (c) PAT recording from the contralateral (non-occluded) finger.

The primary study outcome was degree of deterioration in endothelial function following a high-fat meal. The endothelial function parameter used in this study is called the reactive hyperemia peripheral arterial tonometry (RH-PAT) index. RH-PAT is a noninvasive technique utilizing finger plethysmography to measure pulse volume changes in a digit after stimulating increased blood flow to the digit, termed reactive hyperemia. The change in digital pulse volume during reactive hyperemia relative to baseline is termed the RH-PAT index (see Figure 1). Assessment of endothelial function with RH-PAT methodology has been validated in several previous studies using the same equipment and protocol used in the current study [18–20]. The measurement equipment used for this purpose (Endo-PAT2000, Itamar Medical Ltd, Caesarea, Israel) comprises a finger probe to assess digital volume changes accompanying pulse waves. A blood pressure cuff was placed on one upper arm (study arm) while the other arm served as a control (control arm). Probes were placed on each hand for continuous recording of the PAT signal. After a 10-min equilibration period, the blood pressure cuff was inflated to suprasystolic pressures for 5 min. The cuff was then deflated, while PAT recording was continued for 10 min. The data produced were analysed by a computer in an operator-independent manner. As a measure of digital pulse volume, the RH-PAT index was calculated as the ratio of the average amplitude of the PAT signal over a one-minute time interval, starting one minute after cuff deflation, divided by the average amplitude of the PAT signal of a 3.5-min time period before cuff inflation (baseline). Subsequently, RH-PAT index values from the study arm were normalized to the control arm.

The RH-PAT index was measured before starting study supplement (baseline value) and after completion of each 4-week study period. There was a 2-week wash-out interval between each 4-week study period to avoid possible residual effects from the study supplement. On each occasion, the first measurement was made after an overnight fast. The participant then consumed a high-fat meal, and a second measurement was made 3 h later. The high-fat meal was prepared from microwavable commercial available products and consisted of one cheese omelet (Jimmy Dean Three Cheese Omelet), one croissant (Jimmy Dean Croissant with Egg and Cheese), one 4.75 oz serving of French fries (Ore Ida Extra Crispy Easy Fries) and one bottled water. This meal provided a total of 1010 calories, 56 g of total fat, 15 g of saturated fat, and 225 mg of cholesterol.

Calculation of the required sample size was based on expected differences between the OPC-3 and placebo treatment groups in the percent decrease in RH-PAT index after the fatty meal. The only prior studies available for comparison used an entirely different technology to measure endothelial function, but have shown a difference in percent change between a flavonoid supplement and placebo of between 20 and 50%. For this study, differences of 60% (±30%) and 30% (±15%) in the placebo and supplement treatment groups, respectively, were used for a net between-group difference of 50%. Assuming statistical significance (**α**) of 0.05 and a study sample of 20, nominal study power was estimated at *∼*80%.

Baseline characteristics are reported as means and standard deviations, or frequencies and percents. Tonometry values and before-after percent changes are expressed as means and standard deviations. Unpaired *t*-tests were used to assess between-group differences at each baseline and follow-up interval. Paired *t*-tests were used to compare the difference in percent change between the two study phases. In all cases, **α** = 0.05 was used to determine statistical significance, and the SAS System (SAS Institute, Cary, NC) was used for all analyses.

## 3. Results

### 3.1. Study Group Comparisons

Twenty-three of 25 participants (mean age 43.4 ± 10.4, 78% female) completed all evaluations and are included in this report. There were no smokers. Blood pressures and fasting lipid values were obtained at the baseline visit to confirm there were no significant dyslipidemias or hypertension, since these risk factors for vascular disease are associated with abnormal endothelial function (Table 1). The fasting RH-PAT index at baseline was 2.13 mm (95% CI [1.86, 2.40]) in the active phase and 2.04 mm (95% CI [1.77, 2.31]) in the placebo phase, and were not significantly different (paired *t*-test *P* = .6447). Fasting RH-PAT index values at the end of each 4-week study phase were also not significantly different.

### 3.2. Within-Phase RH-PAT Changes

**Figure 2:**
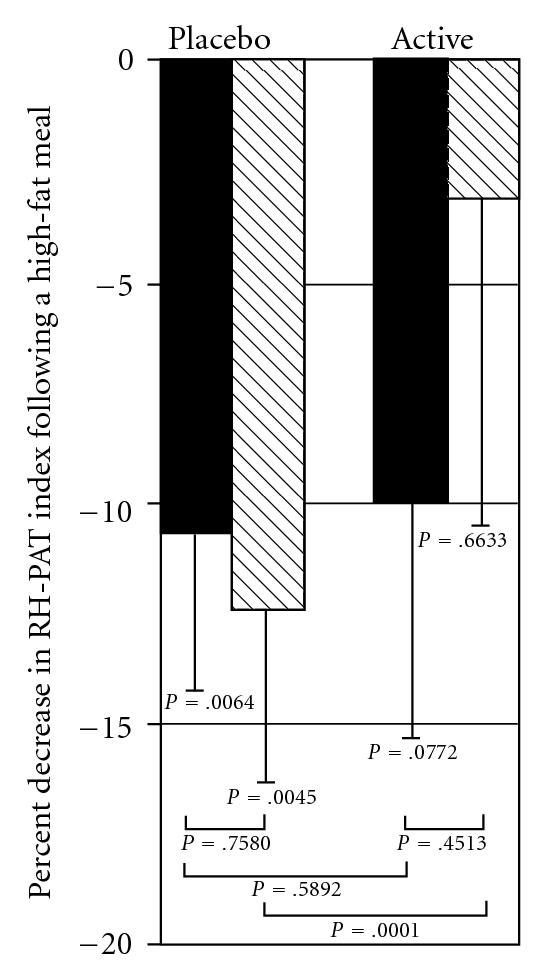
Percent changes in RH-PAT index from a fasting state obtained 3 h after a high-fat meal, at the beginning and end of each study phase. Solid bars, Baseline (before study supplement); Hatched bars, After 4 weeks of study supplement; Values shown as Mean & Standard error.

The percent change in RH-PAT index values between fasting and post-prandial states, which is the parameter of endothelial function deterioration used in this study, are shown in Figure 2. The percent change in the placebo phase at baseline was −10.7 (95% CI [−18.1%, −3.3%]) (*P* = .0064) and at 4 weeks was −12.4 (95% CI [−20.5%, −4.3%]) (*P* = .0045). This indicates that there was endothelial function deterioration after the high-fat meal both at baseline and after 4 weeks of supplementation with the placebo. The percent change in the active phase at baseline was −9.9 (95% CI[−21.1%, −1.2%]) (*P* = .0772), similar to the placebo phase baseline deterioration. After 4 weeks of flavonoid supplementation however there was no statistically significant difference in percent change between fasting and post-prandial values, −3.2 (95% CI [−18.0%, 11.7%]) (*P* = .6633), suggesting possible protection from the flavonoid.

### 3.3. Between-Phase RH-PAT Changes

We then took the percent change in fasting and post-prandial RH-PAT index values and compared the difference in those values between the placebo and active phases. At baseline there was no difference (*t*-test *P* = .5892), but after 4 weeks of supplementation the difference between placebo and active phases was highly statistically significant (*t*-test *P* < 0.0001). Lastly, we assessed the within-group difference in percent change from baseline to 4 weeks and showed no statistically significant difference in either group (paired *t*-test *P* = .7580 for placebo, *P* = .4513 for active). The difference in average percent change between baseline and 4 weeks in the two study phases was not statistically significant (*t*-test *P* = .4492). There were no significant differences by study phase sequence.

## 4. Discussion

Flavonoids are plant secondary metabolites with a wide range of biological effects. A protective role against cardiovascular disease is highly plausible, based on the bulk of epidemiologic data as well as experiments in animal models revealing anti-atherosclerotic effects [1–4]. The epidemiologic studies however have been quite inconsistent. For example, in an elderly Dutch population, in which the primary flavonoid dietary sources were tea, apples and onions, the tertile with the highest consumption of flavonoids had a 68% lower risk for cardiovascular disease [7]. Several studies in the UK and US on the other hand, have demonstrated no relationship between flavonoid intake and cardiovascular risk [8–11]. Many plausible explanations have been offered to explain the discrepancies, all illustrating the extreme difficulty in studying the effect of individual foods and/or their constituents in epidemiologic studies, as well as the biological complexity of the polyphenol class of phytochemicals. Flavonoids in particular have marked heterogeneity in their molecular structures, differences in composition among various foods, differences in amount within the same foods and highly variable bioavailability [12]. Our knowledge is therefore still too limited to formulate recommendations for flavonoid intake to reduce risk of cardiovascular disease.

One way of dealing with this insufficient knowledge is to issue a general recommendation to increase consumption of fruits and vegetables. Even though we do not know the optimal intake or the mechanism by which such a dietary change might reduce cardiovascular risk, this represents a reasonable and well-founded effort to improve the nutritional habits of the general population. Changing dietary habits however is very difficult. Many individuals are likely to never consume adequate amounts of the foods which could reduce their risk for cardiovascular disease. One option is to make up this deficit through supplementation with the food micronutrients and constituents proven to have beneficial physiologic vascular effects. Demonstrating such effects requires the study of specific commercially available products, recently shown for example with a juice powder concentrate [25]. Another option for some individuals would be to supplement their diet with an amount and mixture of flavonoids proven to have a beneficial effect on a valid marker of cardiovascular health.

The majority of studies assessing flavonoid effects on endothelial function have used whole foods. For example, studies testing red wine [26, 27], grape juice [28], black tea [29, 30], soy [31, 32] and cocoa [24], have shown a net beneficial effect on endothelial function as tested by flow-mediated dilation of the brachial artery. It would appear that high flavonoid consumption consistently produces improvement in endothelial function, but it is uncertain how much this perception is the result of publication bias (i.e. studies revealing no benefit are much less likely to be disseminated in the scientific literature) and how much this beneficial effect is dependent on specific types, mixtures and amounts of flavonoid compounds. We therefore chose to study a specific flavonoid supplement with verifiable quality control in the manufacturing process.

Our results indicate that the flavonoid supplement OPC-3 can reduce the acute impairment in endothelial function caused by a high-fat meal. These findings do not imply that all the detrimental effects of a high saturated fat meal are neutralized by taking a flavonoid supplement. The standardized high-fat meal however is a convenient, reproducible means to induce endothelial impairment in healthy volunteers who otherwise have normal endothelial function as proven by their normal fasting values (RH-PAT indices from 1.95 to 2.13 in all subgroups). This same model for inducing endothelial dysfunction in healthy volunteers has been used and validated in other studies assessing effects of food on vascular function [33–38].

Our study results add to a growing literature on the potential benefit of antioxidant phytochemicals providing a long-term effect on endothelial function, in that the flavonoid supplement mitigated the adverse effect of a high-fat meal even though it was not administered immediately prior to the meal. This finding is also consistent with a couple of other studies evaluating long-term ingestion of high-flavonoid foods. Long-term ingestion of a 40% fat diet impaired endothelial function, but this effect was prevented with daily ingestion of red wine [38]. Similar results were reported in coronary heart disease patients after ingestion of grape juice for two weeks [39].

Nitric oxide (NO) is the primary mediator of endothelial-dependent vasodilation [40]. There is a good experimental evidence indicating that the favourable impact of flavonoid supplementation is due to preservation of NO bioactivity by facilitating production of NO via endo thelial NO synthase (eNOS) [41, 42].

There are a couple of limitations to our study. Although we did demonstrate a statistically significant difference in endothelial function deterioration between placebo and flavonoid groups after 4 weeks of supplementation (the main endpoint), the difference in endothelial function deterioration comparing the baseline and 4-week values in the flavonoid arm did not reach statistical significance. This was due to the larger than anticipated variability in deterioration of endothelial function after a high-fat meal. In retrospect, since all of our subjects were free from major cardiac risk factors, a high degree of resilience to a high fat load in some individuals is not surprising. On the other hand, in spite of the healthy population our high-fat meal produced a statistically significant deterioration compared to fasting in the placebo group both at their baseline evaluation and after 4 weeks of supplementation. The same response was demonstrated in the flavonoid group at baseline. In other words, the only group which did not show a deterioration of endothelial function was the flavonoid group after 4 weeks of supplementation. Thus we doubt that the lack of statistical difference in one of our endpoints invalidates the conclusion that the flavonoid supplement mitigated the expected endothelial function deterioration.

The other limitation noted is that we did not make a systematic effort to control for menstrual cycle effects on endothelial function. Although this factor did not appear to impact our results there is literature indicating that baseline endothelial-dependent vasodilatation is slightly larger in the follicular and luteal phases of the cycle compared to the menstrual phase [43]. Effects of the menstrual cycle phase on vasodilatory response to a high-fat meal has not been specifically studied.

In conclusion, we have shown that a high-fat meal on average causes a temporary deterioration in endothelial function, consistent with other published studies. In healthy volunteers 4 weeks of supplementation with a flavonoid supplement significantly blunted this deterioration, compared to the placebo, suggesting that this flavonoid supplementation has a beneficial effect on vascular function in at least certain populations. Whether similar protection of endothelial function would occur in higher risk groups (e.g., those with risk factors for cardiovascular disease such as hypercholesterolemia or diabetes) needs to be studied. The impact of other mitigating factors, such as background consumption of fruits and vegetables, or variable flavonoid intake, also needs to be addressed in future research.

## Figures and Tables

**Table 1:** Baseline demographic characteristics of participants.

Participant profile	Mean ± SD
Age (years)	43.4 ± 10.4
Female (%)	78
Systolic BP	120.8 ± 10.7
Diastolic BP	75.6 ± 5.9
Total cholesterol	193.3 ± 37.4
Triglycerides	71.6 ± 32.7
HDL-C	48.1 ± 16.4
LDL-C	131.0 ± 36.9

BP, blood pressure; HDL-C, high-density lipoprotein cholesterol; LDL-C, low-density lipoprotein cholesterol.
